# *Morinda officinalis* Oligosaccharides Protect Against LPS-Induced Uterine Damage and Endometrial Inflammation in Mice and Bovine Endometrial Epithelial Cells

**DOI:** 10.3390/ani15091286

**Published:** 2025-04-30

**Authors:** Shiwen He, Beibei Yu, Tingting Yu, Tingting Jiang, Diqi Yang, Hui Peng

**Affiliations:** 1 School of Tropical Agriculture and Forestry, Hainan University, Haikou 570228, China; 2 Terrestrial Wildlife Rescue and Epidemic Diseases Surveillance Center of Guangxi, Nanning 530025, China

**Keywords:** *Morinda officinalis* oligosaccharides, endometritis, BENDs, calcium signaling, inflammation

## Abstract

Endometritis poses a significant challenge to the dairy industry, impairing bovine reproductive performance and causing substantial economic losses. Although *Morinda officinalis* oligosaccharides (MOO) exhibit anti-inflammatory properties, their therapeutic potential against endometritis remains unclear. This study investigated MOO’s protective effects against LPS-induced uterine injury in mice and inflammation in bovine endometrial epithelial cells (BENDs), and explored the underlying mechanisms. In mice, MOO attenuated uterine inflammation by improving histopathology, reducing pro-inflammatory cytokines and decreasing oxidative stress. In BEND cells, MOO alleviated LPS-induced inflammation, oxidative stress, and apoptosis via downregulating pro-inflammatory mediators (IL-1β, IL-6, TNF-α, IL-8, TLR4, RELA), restoring antioxidant enzymes (HMOX1, NQO1, Nrf2, NOX4), and modulating apoptosis markers (BAX, cleaved CASP3, CASP9, BCL2). MOO reduced ROS accumulation, preserved mitochondrial membrane potential, and inhibited calcium influx. Critically, the calcium channel agonist Bay K 8644 reversed MOO’s protective effects, confirming calcium signaling modulation as a key mechanism. This study provides the first evidence that MOO mitigates LPS-induced uterine damage and BENDs inflammation through calcium signaling regulation, suggesting its potential for treating inflammation-related reproductive disorders in livestock.

## Simple Summary

Endometritis severely impacts dairy productivity by compromising bovine reproductive health. This study elucidates the therapeutic potential of *Morinda officinalis* oligosaccharides (MOO) against lipopolysaccharide (LPS)-induced uterine injury. In murine models, MOO attenuated uterine inflammation by improving histopathology, suppressing pro-inflammatory cytokines, and mitigating oxidative stress. In bovine endometrial epithelial cells (BENDs), MOO counteracted LPS-induced inflammation, oxidative stress, and apoptosis through three synergistic mechanisms: (1) downregulation of inflammatory mediators, (2) restoration of antioxidant defenses, and (3) modulation of apoptosis-related pathways. MOO further preserved mitochondrial function and inhibited calcium overload. Mechanistic validation using the calcium channel agonist Bay K 8644 confirmed that MOO’s protective effects depend on calcium signaling regulation. These findings establish MOO as a novel regulator of calcium homeostasis, offering a promising strategy to manage inflammatory reproductive disorders in livestock.

## 1. Introduction

Postpartum uterine inflammation is a significant threat to dairy farms, affecting approximately 50% of all dairy cows and resulting in considerable negative impacts on both embryo implantation rates and milk production [1]. Economic analyses reveal that each clinical endometritis case costs $267–$410 per cow, driven by antibiotic therapy, milk yield loss, and premature culling [2]. Untreated cases exacerbate losses to $250 per cow due to infertility and extended calving intervals [3]. During the processes of mating and parturition, the endometrium becomes susceptible to bacterial invasion, which can lead to endometritis. This condition often results in infertility, premature culling of affected cows, and substantial economic losses for dairy operations. The use of pharmaceuticals to treat endometritis renders the milk unsuitable for consumption. Additionally, endometritis leads to decreased pregnancy rates, which is a primary reason for the premature culling of affected cows, further exacerbating economic losses [4]. Key pathogens implicated in endometritis include *Escherichia coli*, *Staphylococcus aureus*, and *Streptococcus* species [5]. The primary therapeutic approach for bovine endometritis has been the use of antibiotics. However, antibiotic treatment poses significant challenges due to the rising issue of drug resistance and residual drug presence in milk, which constitute major risks for the dairy industry. In contrast, Chinese herbal medicine has gained traction in clinical applications due to its safety profile and ease of administration. Importantly, traditional Chinese medicinal approaches target the enhancement of the host’s immune response rather than directly attacking pathogens, thus mitigating the development of bacterial resistance [6].

Bovine endometrial epithelial cells (BENDs) serve as a vital defense against pathogenic microorganisms, forming a critical barrier between potential pathogens in the uterine lumen and the deeper uterine tissues. This barrier is essential for controlling permeability and regulating inflammation [7]. Key pathogens implicated in endometritis include *Escherichia coli*, *Staphylococcus aureus*, and *Streptococcus* species. LPS derived from *E. coli* was selected as the primary inflammatory inducer due to its clinical prevalence in endometritis and its ability to activate TLR4/NF-κB-driven cytokine release in BENDs [8,9]. Calcium functions as a crucial second messenger, with BENDs heavily dependent on fluctuations in intracellular calcium ions and subsequent signaling cascades. During inflammatory responses, calcium signaling in BENDs can be significantly elevated, with intracellular calcium levels rising 5 to 10 times above baseline [10]. Previous studies have demonstrated that LPS can induce calcium channel activity and activate calcium signaling pathways in BENDs [11]. Similarly, research by Liu et al. found that *E. coli* led to abnormal increases in cytoplasmic calcium and mitochondrial dysfunction in primary BENDs, as evidenced by live-cell imaging of calcium reporters [12]. Additionally, recent findings indicate that cows with endometritis have lower plasma calcium concentrations compared to healthy cows [13].

*Morinda officinalis* (MO), along with *Alpinia oxyphylla*, *Amomum villosum*, and *Areca catechu*, is renowned as one of the “Four Southern Medicines”. The roots of MO, a prominent member of the Rubiaceae family, have been utilized extensively in Traditional Chinese Medicine [14]. *Morinda officinalis* oligosaccharides (MOO), the bioactive compounds extracted from MO roots, are known for their use as a Yang-tonic agent to enhance renal function and sexual performance [15,16]. MOO has garnered significant attention primarily for its antidepressant properties, demonstrating the ability to inhibit inflammation, astrocytic dysfunction, and mitochondrial damage in the brain [17]. Additionally, MOO has been shown to effectively mitigate LPS-induced M1 macrophage polarization by regulating HSP90 and NF-κB pathways [18]. Although MO is a natural plant that is widely available and easily accessible in southern China, and its active component MOO exhibits anti-inflammatory and anti-apoptotic properties, its protective effects on ruminant endometritis remain unclear. Emerging studies demonstrate that natural compounds such as polyphenol ellagic acid and acidic cannabinoids suppress inflammation via calcium signaling modulation. By analogy, MOO may alleviate endometrial inflammation through similar calcium-dependent pathways [19,20].

In this study, a mouse model of endometrial inflammatory injury was established to investigate ruminant endometritis. We examined the potential therapeutic effects of MOO on endometrial inflammatory injury using this mouse model and analyzed the underlying regulatory mechanisms through transcriptomic techniques. To delve deeper into the mechanisms by which MOO mitigates endometrial inflammatory injury, we utilized bovine BENDs as an in vitro model.

## 2. Materials and Methods

### 2.1. Experimental Animal Groups and Treatments

**Figure 1:**
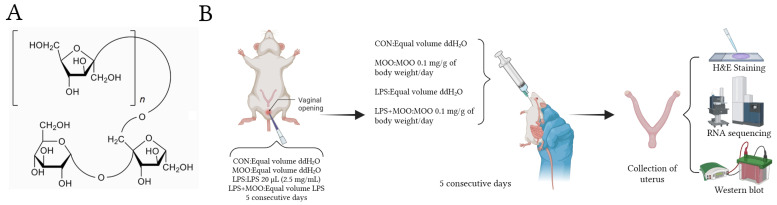
(**A**) Chemical structure of *Morinda officinalis* oligosaccharides (MOO). (**B**) Schematic diagram of the animal experiment.

Specific-pathogen-free (SPF) grade Kunming female virgin mice, approximately 8 weeks old, were procured from Haikou Tonghui Biotechnology Co., Ltd. (ethical approval number: HNUAUCC-2023-00032). The mice were maintained in a temperature-controlled environment (24 ± 1 °C) with a 12 h light/dark cycle and provided with unrestricted access to food and water. They were allowed a one-week acclimatization period prior to the experiment. All procedures adhered to the Institutional Animal Care and Use Committee guidelines of Hainan University. Mice, weighing around 25 g, were randomly assigned to four groups (*n* = 6 per group): control, LPS, MOO, and LPS + MOO groups. The endometritis model in mice was established following previously described methods [9]. For groups requiring LPS treatment, LPS (20 μL, 2.5 mg/mL, Sigma, St. Louis, MO, USA) was administered intrauterinely for five consecutive days to induce endometrial inflammatory injury [8,21]. At the same time, mice in the MOO treatment groups received MOO (0.1 mg/g of body weight/day, 50% purity; Tongrentang Co., Ltd., Beijing, China) via gavage for five days. Control mice and those not receiving LPS or MOO were given an equivalent volume of ddH_2_O. All mice were euthanized 24 h after the final MOO administration (Figure 1).

### 2.2. H and E Staining

The uterine tissues were fixed overnight and subsequently embedded in paraffin. Paraffin-embedded uterine sections were then dehydrated through a series of alcohol solutions (100%, 95%, and 90%) and stained with hematoxylin and eosin (H and E). The stained sections were examined using an optical microscope (Olympus, Shinjuku-ku, Tokyo, Japan).

### 2.3. RNA Sequencing and Bioinformatic Analysis

RNA sequencing was carried out by Biomarker Technologies Co., Ltd. (Beijing, China). Total RNA was extracted using TRIzol reagent (Invitrogen, Waltham, MA, USA), and RNA integrity was verified via Agilent 2100 Bioanalyzer (RIN > 8.0). cDNA libraries: After quality control of the libraries, different libraries were constructed using the NEB NextUltra II RNA Library Prep Kit (New England Biolabs, Ipswich, MA, USA). Library quality was assessed using the Agilent 2100 Bioanalyzer (Agilent Technologies, Santa Clara, CA, USA; insert size: 300 ± 50 bp; concentration > 2 nM). Libraries were pooled in randomized order and sequenced on the Illumina NovaSeq 6000 platform with 150 bp paired-end reads. Quality Control Metrics: (1) Sequencing Depth: An average of 40 million reads per sample (range: 38–42 million) was achieved. (2) Alignment Rate: 96.24–96.59% of reads were mapped to the mouse reference genome (GRCm39) using using BMKCloud’s (Biomark Corporation, Hong Kong, China) optimized STAR aligner. Differentially expressed genes (DEGs) were identified using DESeq2 with thresholds of |log_2_(fold change)| > 1 and an adjusted *p*-value < 0.05 (Benjamini–Hochberg correction). GO and KEGG pathway analyses were conducted using clusterProfiler (v4.6.0). Raw sequencing data are available at the China National center for Bioinformation under accession code PRJCA032167.

### 2.4. RNA Extraction and Real-Time Quantitative PCR

Total RNA was isolated using TRIzol reagent (TaKaRa Bio, Inc., Dalian, China), and cDNA synthesis was carried out with ABScript II RT Master Mix for qPCR (ABclonal Biotechnology, Wuhan, China). The primer sequences used are provided in Supplementary Table S1. Real-time quantitative PCR was conducted with ABclonal 2X Universal SYBR Green Fast qPCR Mix (ABclonal Biotechnology, China). Gene expression levels were quantified using the 2^−ΔΔCt^ method, with GAPDH serving as the normalization control.

### 2.5. Western Blot

Endometrial tissues from mice and BENDs were collected and lysed. For each sample, 30 micrograms of total protein were loaded into a 12% SDS–PAGE gel, followed by electrophoretic separation. Proteins were then transferred onto PVDF membranes (Millipore, Bedford, MA, USA). After blocking, membranes were incubated overnight at 4 °C with primary antibodies: anti-NQO1 (ABclonal A1518, 1:1000), anti-caspase-3 (ABclonal A11040, 1:1000), anti-BCL2 (bioss bs-34012R, 1:1000), anti-HO-1 (proteintech 10701-1-AP, 1:1000), anti-BAX (Beyotime AF1270, 1:1000), anti-TLR4(bioss bs-20594R, 1:1000), anti-RELA (bioss bs-20159R, 1:1000), anti-phosphor-RELA (Ser536) (bioss bs-0982R, 1:1000), and anti-ACTB(ABclonal AC038, 1:10,000). The following day, the membranes were treated with HRP-conjugated secondary antibodies. Protein bands were visualized using Image-Pro Plus 6.0 software (Media Cybernetics, Inc., Silver Spring, MD, USA) and quantified with Quantity One v4.6.6 software (Bio-Rad Laboratories, Hercules, CA, USA).

### 2.6. Cell Culture and Drug Treatment

BENDs were obtained from Beina Biotechnology Co., Ltd. (BNCC; Beijing, China; catalog number 359233). BENDs were cultured in six-well plates in DMEM/F-12 medium supplemented with 10% fetal bovine serum (FBS, AusgeneX, Loganholme, Queensland, Australia) at 37 °C under 5% CO_2_. Once the cells reached 70–80% confluence, they were treated with 1 μg/mL LPS for 12 h. Subsequently, different concentrations of MOO (5, 50, and 500 μg/mL) were added to the BENDs and incubated for 12 h. In the Bay K 8644 (MedChemExpress (Junction, NJ, USA), HY-0588) group, 2 μM Bay K 8644 was administered to the BENDs 2 h before the addition of MOO [22,23].

### 2.7. Measurement of Cell Viability

The cytotoxicity of MOO was evaluated using a CCK-8 assay (Beyotime, Haimen, China). BENDs were seeded in 96-well plates and incubated with MOO at concentrations of 0, 5, 50, 500, 1000, 2500, and 5000 μg/mL for a 12 h period.

### 2.8. Assessment of Reactive Oxygen Species

BENDs were seeded into 24-well plates and treated with LPS, MOO, or a combination of both. Following treatment, cells were incubated with 10 μM 2,7-dichlorodihydrofluorescein diacetate (DCFH-DA, Beyotime, China) for 20 min. Subsequently, cells were stained with DAPI as per the manufacturer’s instructions. Fluorescence was then observed using a fluorescence microscope (Nikon Inc., Melville, NY, USA).

### 2.9. Detection of Mitochondrial Membrane Potential

The mitochondrial membrane potential (MMP) in BENDs was assessed using a JC-1 kit (Beyotime, China). BENDs were plated into 24-well plates and treated with LPS, MOO, or their combination. After treatment, a JC-1 working solution (0.6 mL) was added to each well, and the cells were incubated for 20 min at 37 °C. Post-incubation, cells were rinsed three times with phosphate-buffered saline [24]. The fluorescence intensity was then examined using a fluorescence microscope (Nikon Inc., Melville, NY, USA).

### 2.10. Measurement of Intracellular Ca^2+^

BENDs were incubated with 5 μM Fluo-4 AM (Beyotime, China), a Ca^2+^ sensitive dye, at 37 °C for 15 min. Post-incubation, cells underwent three washes to eliminate any unbound dye and were subsequently incubated for an additional 30 min at 37 °C to ensure full de-esterification of intracellular AM esters. Thereafter, cells were stained with 1 μg/mL Hoechst 33342 for 10 min to mark the cell nuclei, followed by three washes to remove any unbound stain. Finally, cells were maintained in serum-free medium to facilitate the measurement of intracellular calcium levels [25]. The fluorescence intensity, indicative of intracellular Ca^2+^ concentration, was assessed using a fluorescence microscope.

### 2.11. Statistical Analysis

Unless otherwise specified, all data are presented as the mean ± SEM. Statistical analysis was performed using SPSS version 27 (IBM-SPSS Inc., Chicago, IL, USA). Variables were analyzed using one-way ANOVA with least significant difference (LSD) post hoc comparisons. Statistical significance was defined as *p* < 0.05.

## 3. Results

### 3.1. MOO Alleviates LPS-Induced Uterine Tissue Damage in Mice

**Figure 2:**
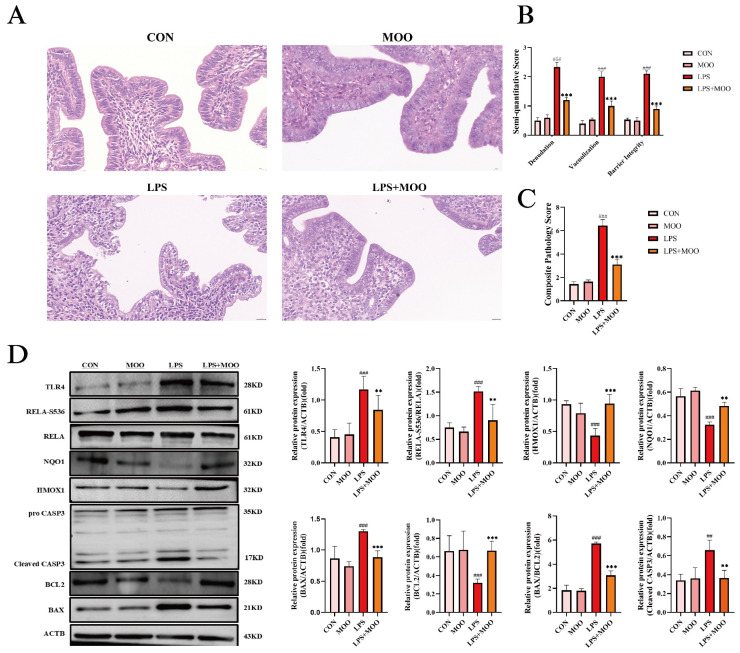
MOO alleviates LPS-induced uterine tissue damage in mice. (**A**) Morphological changes in the uterine tissue of mice. Scale bar = 20 μm. (**B**) Semi-quantitative scores for epithelial denudation, vacuolization, and barrier integrity. (**C**) Composite pathology scores across experimental groups. (**D**) Western blot analysis of inflammation, oxidative stress, and apoptosis-related protein expression in uterine tissues. ##, *p* < 0.01 and ###, *p* < 0.001 vs. the control group; **, *p* < 0.01 and ***, *p* < 0.001 vs. the LPS group. Data are presented as the mean ± SEM (*n* = 3).

To evaluate the protective effects of MOO on uterine tissue, we first examined the histological changes in the mouse uterus. As shown in Figure 2A, the uterine tissues in the control (CON) and MOO groups displayed an intact mucosal epithelial structure, with tightly arranged cells and normal morphology, and no evident edema or inflammatory cell infiltration. In contrast, the LPS group exhibited severe epithelial denudation, widespread intracellular vacuolization, and disrupted barrier integrity, accompanied by structural disorganization. Histopathological scoring based on a validated semi-quantitative system confirmed that LPS challenge induced significant endometrial damage across all three pathological markers (*p* < 0.001 vs. CON) [26]. Notably, co-treatment with MOO (LPS + MOO group) markedly attenuated these pathologies, significantly reducing the severity of epithelial denudation, vacuolization, and barrier disruption compared to the LPS group (*p* < 0.001; Figure 2B,C).

Next, we analyzed the expression of proteins associated with inflammation, oxidative stress, and apoptosis in the uterine tissues. Western blot analysis revealed that LPS treatment significantly upregulated the expression of key inflammatory proteins, including TLR4 and phosphorylated RELA at Ser536 (RELA-S536), while downregulating the expression of the oxidative stress-related proteins quinone oxidoreductase-1 (NQO1) and heme oxygenase-1 (HMOX1). Furthermore, LPS treatment activated pro-apoptotic proteins cleaved CASP3 and BAX while suppressing the expression of the anti-apoptotic protein BCL2. As expected, MOO treatment mitigated the LPS-induced activation of inflammatory markers TLR4 and RELA-S536, as well as the pro-apoptotic proteins cleaved CASP3 and BAX. Concurrently, it restored the expression of NQO1, HMOX1, and the anti-apoptotic protein BCL2 (Figure 2D). These findings suggest that MOO effectively alleviates LPS-induced uterine inflammation and tissue damage in mice.

### 3.2. Transcriptomic Analysis of the Effects of MOO on LPS-Induced Uterine Injury in Mice

**Figure 3:**
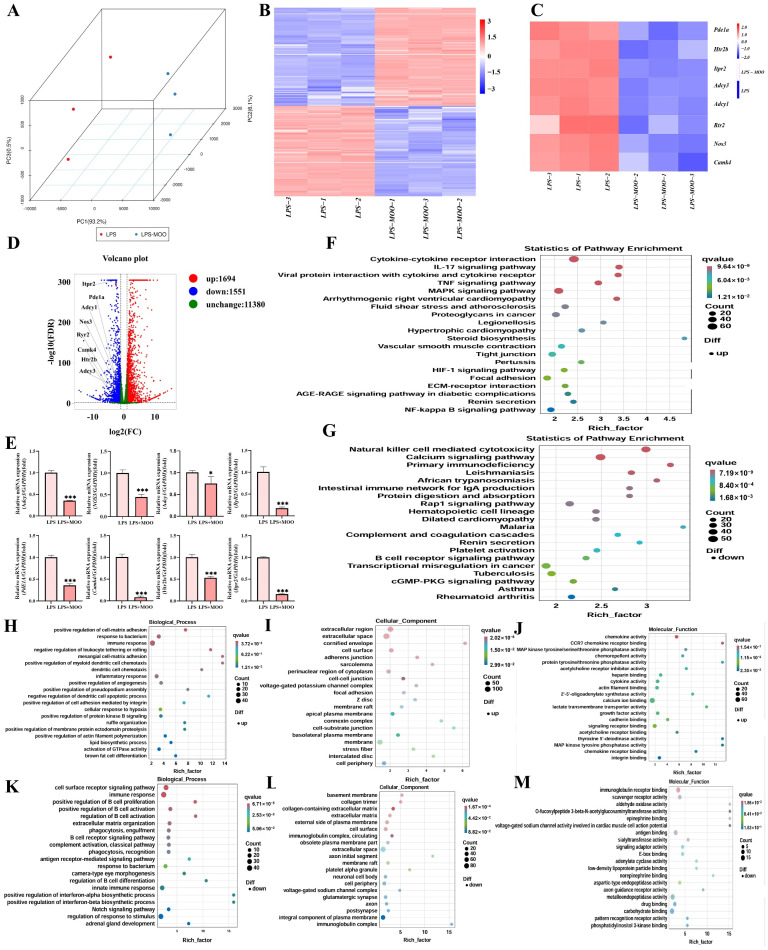
Transcriptomic analysis of the effects of MOO on LPS-induced uterine injury in mice. (**A**) Principal component analysis (PCA) of uterine tissue samples from the LPS and LPS + MOO groups. Red dots represent the LPS group, while blue dots represent the LPS + MOO group. (**B**) Heatmap of differentially expressed genes (DEGs) in the uterine tissue. (**C**) Heatmap showing DEGs involved in calcium signaling. (**D**) Volcano plot of DEGs. Red indicates upregulation, blue indicates downregulation, and green indicates no significant change. (**E**) Validation of transcriptomic data by real-time PCR. Data are presented as the mean ± SEM (* *p* < 0.05; *** *p* < 0.001). (**F**,**G**) KEGG pathway enrichment analysis of upregulated and downregulated DEGs, respectively. (**H**–**J**) GO classification enrichment analysis of upregulated DEGs in the Biological Process, Molecular Function, and Cellular Component categories, respectively. (**K**–**M**) GO classification enrichment analysis of downregulated DEGs in the Biological Process, Molecular Function, and Cellular Component categories, respectively.

To further explore the protective effects of MOO on the uterus, transcriptomic differences between the LPS and LPS + MOO groups were analyzed. Principal component analysis (PCA) demonstrated clear separation between the two groups, indicating distinct gene expression profiles (Figure 3A). Transcriptomic analysis identified 1694 overexpressed and 1551 underexpressed differentially expressed genes (DEGs) in the LPS + MOO group compared to the LPS group (Figure 3B). Kyoto Encyclopedia of Genes and Genomes (KEGG) pathway enrichment analysis revealed that upregulated DEGs in the LPS + MOO group were significantly enriched in pathways such as cytokine–cytokine receptor interaction (Figure 3F), whereas downregulated DEGs were predominantly associated with the calcium signaling pathway. Gene Ontology (GO) enrichment analysis further showed that, in biological processes, upregulated DEGs were enriched in terms related to immune response and response to bacterium (Figure 3H), while downregulated DEGs were associated with immune response and positive regulation of B cell proliferation (Figure 3K). In the cellular component category, upregulated DEGs were enriched in extracellular region and cell surface (Figure 3I), whereas downregulated DEGs were enriched in the basement membrane (Figure 3L). For molecular function, upregulated DEGs were enriched in chemokine activity and CCR7 chemokine receptor binding (Figure 3J), while downregulated DEGs were enriched in immunoglobulin receptor binding (Figure 3M). Notably, several key genes involved in the calcium signaling pathway, including *PDE1A*, *RyR2*, *Adcy3*, *Adcy1*, *Htr2b*, *Itpr2*, *Camk4*, and *Nos3*, were significantly downregulated in the LPS + MOO group compared to the LPS group (Figure 3C,D). To validate these findings, qPCR analysis was performed on the eight selected genes, and the results were consistent with the transcriptomic data (Figure 3E). These results suggest that MOO alleviates LPS-induced uterine injury, potentially through the inhibition of the calcium signaling pathway.

### 3.3. MOO Alleviates LPS-Induced Inflammation in BENDs

**Figure 4:**
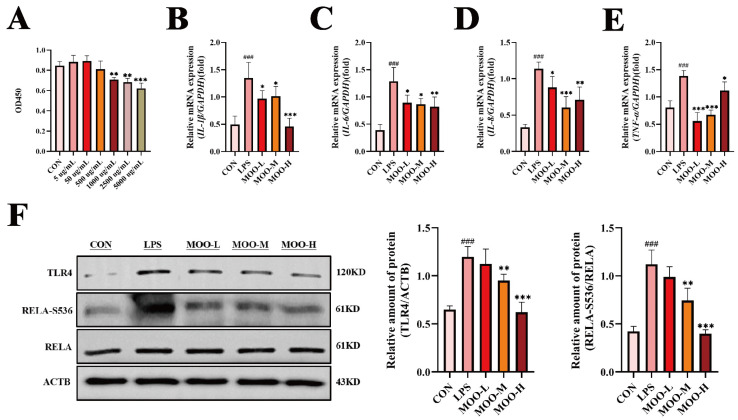
MOO alleviates LPS-induced inflammation in BENDs. (**A**) Cell viability of BENDs treated with MOO at various doses for 12 h, determined using the CCK-8 assay. **, *p* < 0.01 and ***, *p* < 0.001 vs. the control (**C**) group. (**B**–**E**) qPCR analysis of the effects of different MOO doses on inflammatory cytokine expression. (**F**) Western blot analysis of inflammation-related protein expression in BENDs treated with various doses of MOO. ###, *p* < 0.001 vs. the control group; *, *p* < 0.05, **, *p* < 0.01 and ***, *p* < 0.001 vs. the LPS group. Data are presented as the mean ± SEM (*n* = 3).

Based on the transcriptomic findings suggesting MOO modulates immune responses, we further investigated its effects on LPS-induced inflammation in BENDs. First, the cytotoxicity of MOO was evaluated using a CCK-8 assay. As shown in Figure 4A, MOO at concentrations of 5, 50, and 500 μg/mL had no significant effect on cell viability, whereas concentrations of 1000 μg/mL and above significantly inhibited cell viability in a dose-dependent manner. Therefore, MOO concentrations of 5 μg/mL (low, MOO-L), 50 μg/mL (medium, MOO-M), and 500 μg/mL (high, MOO-H) were selected for subsequent experiments. Next, the effects of MOO on LPS-induced pro-inflammatory cytokine expression were examined. qPCR analysis revealed that MOO-L, MOO-M, and MOO-H treatments significantly reduced the LPS-induced mRNA levels of the pro-inflammatory cytokines *IL-1β*, *IL-6*, *IL-8*, and *TNF-α* (Figure 4B–E). Additionally, the expression levels of key inflammatory proteins were assessed via Western blot. LPS treatment significantly upregulated the expression of TLR4 and phosphorylated RELA at Ser536 (RELA-S536) in BENDs. While MOO-L treatment reduced TLR4 and RELA-S536 expression, the changes were not statistically significant. In contrast, MOO-M and MOO-H treatments markedly suppressed the expression of TLR4 and RELA-S536 in a dose-dependent manner (Figure 4F). These results demonstrate that MOO effectively inhibits LPS-induced inflammation in BENDs in vitro, with higher concentrations exerting more pronounced anti-inflammatory effects.

### 3.4. MOO Alleviates LPS-Induced Oxidative Stress in BENDs

**Figure 5:**
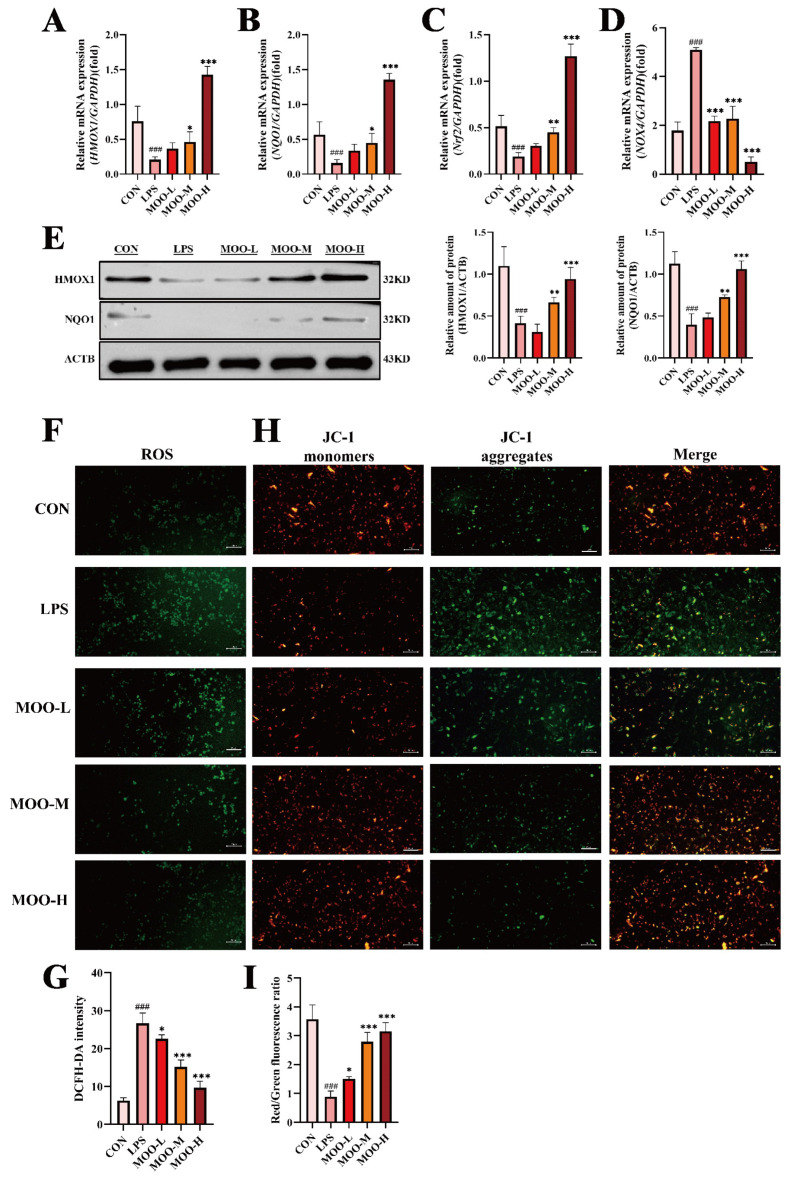
MOO alleviates LPS-induced oxidative stress in BENDs. (**A**–**D**) qPCR analysis of oxidative stress marker expression in BENDs treated with various doses of MOO. (**E**) Western blot analysis of oxidative stress-related protein expression in BENDs treated with MOO at different doses. (**F**,**G**) Effects of different MOO doses on intracellular ROS levels in LPS-treated BENDs. (**H**,**I**) Effects of different MOO doses on mitochondrial membrane potential in LPS-treated BENDs. ###, *p* < 0.001 vs. the control group; *, *p* < 0.05, **, *p* < 0.01 and ***, *p* < 0.001 vs. the LPS group. Data are presented as the mean ± SEM (*n* = 3).

Previous studies have demonstrated that reactive oxygen species (ROS) generated under oxidative stress conditions can influence calcium signaling [27]. Transcriptomic analysis from this study suggested that the downregulation of calcium signaling might be involved in MOO-mediated mitigation of LPS-induced uterine damage. Thus, we investigated whether MOO alleviates LPS-induced oxidative stress in BENDs. As expected, LPS treatment significantly suppressed the mRNA expression of oxidative stress markers, including *HMOX1*, *NQO1*, and *Nrf2*, while upregulating *NOX4* transcription levels (Figure 5A–D). MOO treatment alleviated these changes in a dose-dependent manner, with the high-dose group (MOO-H) showing the most pronounced effects in restoring oxidative stress marker expression (Figure 5A–D). Consistent with the qPCR results, Western blot analysis demonstrated that MOO-M and MOO-H treatments significantly restored the downregulated protein levels of HMOX1 and NQO1 caused by LPS (Figure 5E). To further examine the effects of MOO on ROS production, we employed the fluorogenic probe DCFH-DA to quantify ROS levels in BENDs. LPS treatment led to a substantial accumulation of ROS, while MOO treatment effectively reduced ROS accumulation in a dose-dependent manner (Figure 5F,G). To explore whether MOO-mediated improvements in ROS levels were linked to the mitigation of mitochondrial dysfunction, we assessed mitochondrial membrane potential (MMP, ΔΨm), a key indicator of mitochondrial health, using JC-1 staining. LPS-treated BENDs displayed mitochondrial damage, as indicated by the loss of mitochondrial membrane potential and the predominance of green fluorescent monomers due to JC-1 dissociation (Figure 5H,I). In contrast, MOO treatment markedly increased the red/green fluorescence ratio intensity in a dose-dependent manner, indicating the restoration of mitochondrial membrane potential and reversal of mitochondrial damage (Figure 5H,I). Phase-contrast imaging further confirmed that MOO treatment restored LPS-induced morphological damage in BENDs, with near-complete recovery observed in the high-dose group (Supplementary Figure S1). These results demonstrate that MOO alleviates LPS-induced oxidative stress in BENDs by reducing ROS accumulation and preserving mitochondrial function.

### 3.5. MOO Mitigates LPS-Induced Apoptosis in BENDs

**Figure 6:**
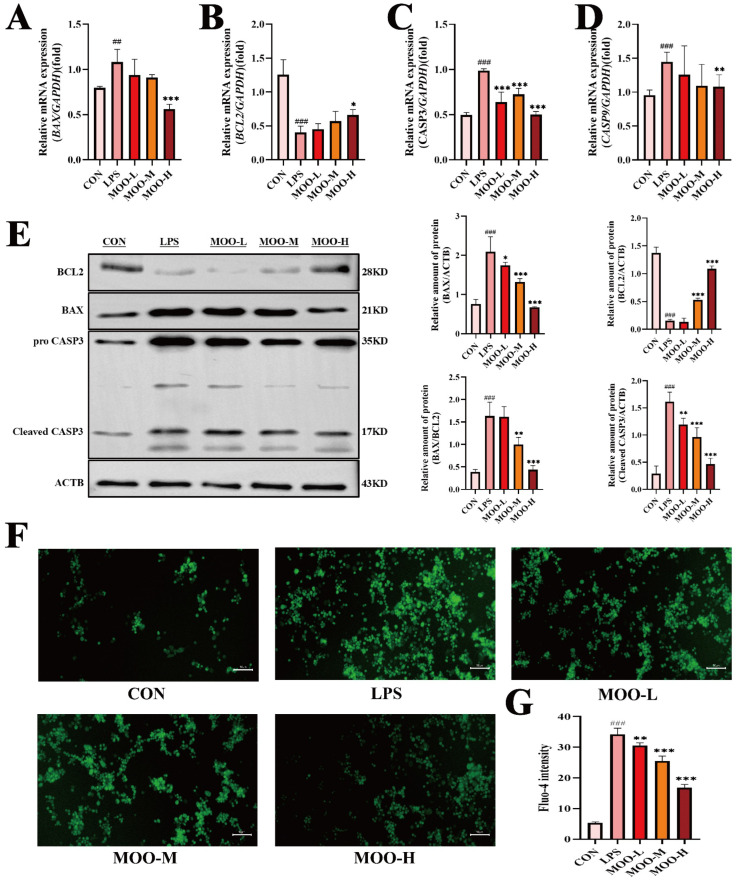
MOO mitigates LPS-induced apoptosis in BENDs. (**A**–**D**) qPCR analysis of apoptosis marker expression in BENDs treated with various doses of MOO under LPS stimulation. (**E**) Western blot analysis of apoptosis-related protein expression in BENDs treated with MOO under LPS stimulation. (**F**,**G**) Effects of different MOO doses on calcium ion levels in LPS-induced BENDs. ##, *p* < 0.01 and ###, *p* < 0.001 vs. the control group; *, *p* < 0.05, **, *p* < 0.01 and ***, *p* < 0.001 vs. the LPS group. Data are presented as the mean ± SEM (*n* = 3).

Endometrial epithelial cell apoptosis is a well-documented consequence of inflammation-induced uterine damage [28,29]. Given our previous findings that MOO alleviates LPS-induced inflammation and oxidative stress in BENDs, we further investigated whether MOO could also reduce LPS-induced apoptosis. qPCR analysis revealed that LPS treatment significantly increased the mRNA levels of pro-apoptotic genes (*BAX*, *CASP3*, and *CASP9*) while reducing the expression of the anti-apoptotic gene *BCL2* (Figure 6A–D). High-dose MOO (MOO-H) effectively countered these effects by suppressing the expression of pro-apoptotic genes and upregulating *BCL2* (Figure 6A–D). To confirm these findings at the protein level, Western blot analysis was conducted. Consistent with the qPCR results, MOO treatment significantly reduced the expression of pro-apoptotic proteins BAX and cleaved CASP3 while increasing BCL2 protein levels, with the most pronounced effects observed in the MOO-H group (Figure 6E). Considering that transcriptomic analysis suggested calcium signaling involvement in MOO-mediated protection against LPS-induced damage, intracellular calcium ion concentration was assessed using Fluo-4 staining. LPS treatment caused a marked increase in intracellular calcium influx in BENDs, whereas MOO significantly attenuated this influx in a dose-dependent manner (Figure 6F,G). These results indicate that MOO effectively inhibits LPS-induced apoptosis in BENDs and mitigates the associated increase in intracellular calcium ion levels.

### 3.6. Activation of Calcium Signaling Impedes the Anti-Inflammatory Effects of MOO in LPS-Treated BENDs

**Figure 7:**
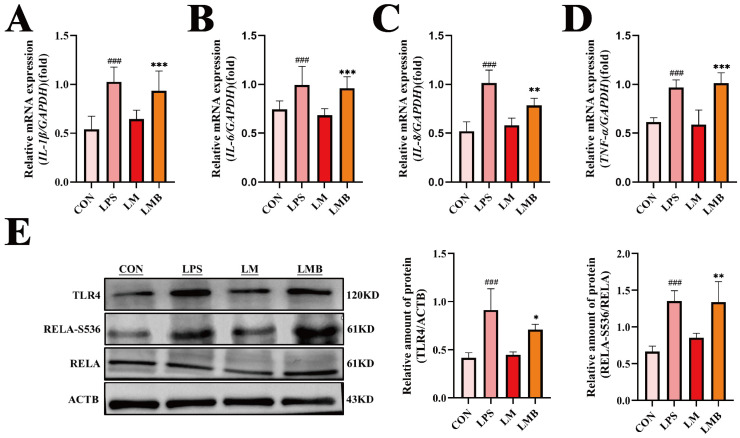
Activation of calcium signaling impedes the anti-inflammatory effects of MOO in LPS-induced BENDs. (**A**–**D**) qPCR analysis of inflammatory cytokine expression in LPS-induced BENDs pretreated with Bay K 8644 under MOO treatment. (**E**) Western blot analysis of inflammation-related protein expression in LPS-induced BENDs treated with MOO and Bay K 8644. ###, *p* < 0.001 vs. the control group; *, *p* < 0.05, **, *p* < 0.01 and ***, *p* < 0.001 vs. the LM group. Data are presented as the mean ± SEM (*n* = 3). LM: LPS + MOO-H; LMB: LPS + MOO - H + Bay K 8644.

Our previous results demonstrated that MOO effectively alleviates LPS-induced inflammation in BENDs, partly by suppressing calcium signaling. To investigate whether calcium signaling mediates the anti-inflammatory effects of MOO, we used Bay K 8644, an agonist of L-type calcium channels, to activate this pathway. qPCR analysis revealed that Bay K 8644 treatment (LMB group: LPS + MOO - H + Bay K 8644) significantly increased the transcription levels of pro-inflammatory cytokines (*IL-1β*, *IL-6*, *IL-8*, and *TNF-α*) compared to the LM group (LPS + MOO-H) (Figure 7A–D). Consistently, Western blot analysis showed that Bay K 8644 pretreatment effectively reversed the suppressive effects of MOO on the expression of TLR4 and RELA-S536 in LPS-treated BENDs (Figure 7E). These findings indicate that calcium signaling activation can hinder the regulatory effects of MOO on inflammation in LPS-induced BENDs, suggesting that calcium signaling is a critical mediator in the anti-inflammatory mechanisms of MOO.

### 3.7. Activation of Calcium Signaling Impairs the Protective Effects of MOO Against LPS-Induced Oxidative Stress in BENDs

**Figure 8:**
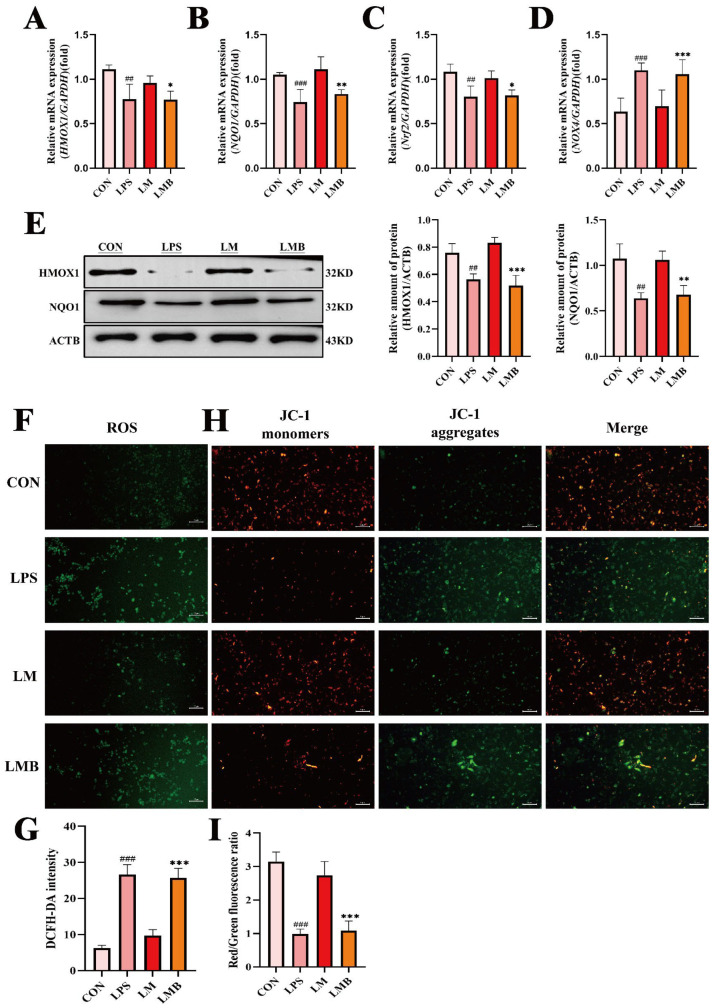
Activation of calcium signaling impairs the protective effects of MOO against LPS-induced oxidative stress in BENDs. (**A**–**D**) qPCR analysis of oxidative stress marker expression in LPS-induced BENDs pretreated with Bay K 8644 under MOO treatment. (**E**) Western blot analysis of oxidative stress-related protein expression in LPS-induced BENDs treated with MOO and Bay K 8644. (**F**,**G**) Effects of Bay K 8644 pretreatment on ROS levels regulated by MOO in LPS-induced BENDs. (**H**,**I**) Effects of Bay K 8644 pretreatment on mitochondrial membrane potential regulated by MOO in LPS-induced BENDs. ##, *p* < 0.01 and ###, *p* < 0.001 vs. the control group; *, *p* < 0.05, **, *p* < 0.01 and ***, *p* < 0.001 vs. the LM group. Data are presented as the mean ± SEM (*n* = 3). LM: LPS + MOO-H; LMB: LPS + MOO - H + Bay K 8644.

To further evaluate the role of calcium signaling in the protective effects of MOO against LPS-induced oxidative stress in BENDs, we examined the impact of Bay K 8644 on oxidative stress markers. As anticipated, qPCR analysis revealed that the expression of key oxidative stress-related genes (*HMOX1*, *NQO1*, *Nrf2*, and *NOX4*) was significantly downregulated in the LMB group (LPS + MOO - H + Bay K 8644) compared to the LM group (LPS + MOO-H) (Figure 8A–D). Consistently, Western blot results confirmed that Bay K 8644 treatment diminished the protein levels of HMOX1 and NQO1 in BENDs co-treated with LPS and MOO (Figure 8E). Additionally, ROS accumulation assays revealed a marked increase in ROS levels in the LMB group compared to the LM group (Figure 8F,G). Similarly, mitochondrial membrane potential (MMP) analysis using JC-1 staining showed a significant rise in green fluorescent monomers in the LMB group, indicating greater mitochondrial dysfunction compared to the LM group (Figure 8H,I). These findings suggest that the activation of calcium signaling disrupts the ability of MOO to alleviate LPS-induced oxidative stress in BENDs, further underscoring the pivotal role of calcium signaling in this regulatory process.

### 3.8. Activation of Calcium Signaling Impairs the Anti-Apoptotic Effects of MOO in LPS-Induced BENDs

**Figure 9:**
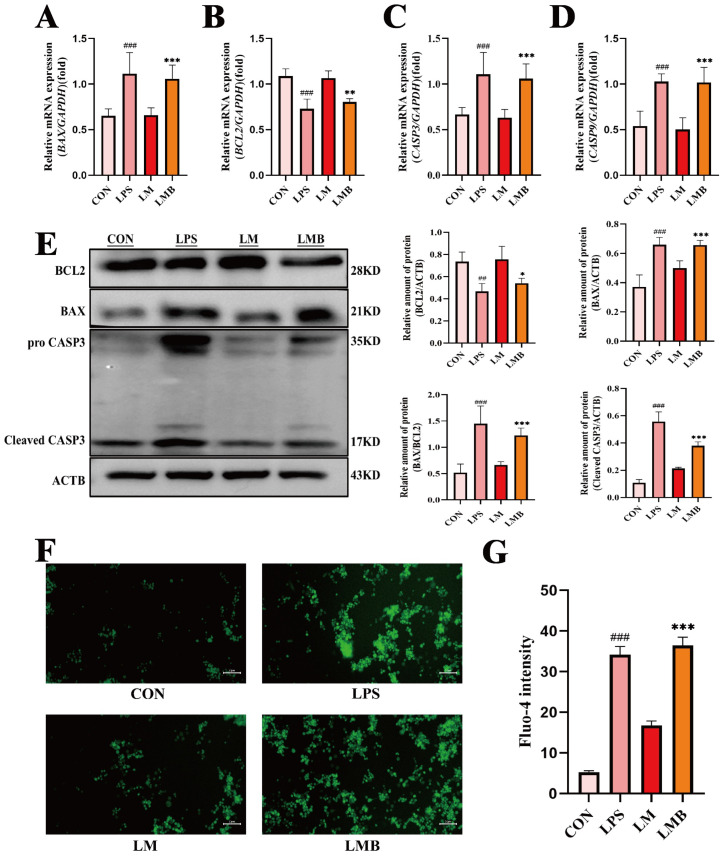
Activation of calcium signaling impairs the anti-apoptotic effects of MOO in LPS-induced BENDs. (**A**–**D**) qPCR analysis of apoptosis marker expression in LPS-induced BENDs pretreated with Bay K 8644 under MOO treatment. (**E**) Western blot analysis of apoptosis-related protein expression in LPS-induced BENDs treated with MOO and Bay K 8644. (**F**,**G**) Effects of Bay K 8644 pretreatment on calcium ion regulation by MOO in LPS-induced BENDs ##, *p* < 0.01 and ###, *p* < 0.001 vs. the control group; *, *p* < 0.05, **, *p* < 0.01 and ***, *p* < 0.001 vs. the LM group. Data are presented as the mean ± SEM (*n* = 3). LM: LPS + MOO-H; LMB: LPS + MOO - H + Bay K 8644.

Given that calcium signaling activation impeded the anti-inflammatory and antioxidative effects of MOO in LPS-induced BENDs, we further explored its impact on apoptosis. qPCR analysis revealed a significant upregulation of pro-apoptotic genes (*BAX*, *CASP3*, and *CASP9*) and a notable suppression of the anti-apoptotic gene *BCL2* in the LMB group (LPS + MOO - H + Bay K 8644) compared to the LM group (LPS + MOO - H) (Figure 9A–D). These results were consistent with Western blot findings, which showed that Bay K 8644 pretreatment increased the protein levels of BAX and cleaved CASP3, while significantly reducing BCL2 protein expression in the LPS and MOO co-treated BENDs (Figure 9E). Furthermore, intracellular calcium ion levels, assessed using Fluo-4 staining, demonstrated a substantial influx of calcium ions into BENDs under calcium signaling activation conditions (Figure 9F,G). These findings indicate that the activation of calcium signaling markedly disrupts the protective effects of MOO against LPS-induced apoptosis in BENDs, highlighting the critical role of calcium signaling in modulating apoptosis under inflammatory conditions.

## 4. Discussion

Endometritis is a significant challenge for the dairy industry, particularly in the postpartum period. The widespread use of antibiotics to treat uterine infections has led to growing concerns over antibiotic resistance and drug residues, compelling researchers and livestock enterprises to seek more sustainable and environmentally friendly alternatives. Traditional Chinese Medicine has been extensively studied for the treatment of bovine endometritis, with promising results that highlight the potential of active compounds in Traditional Chinese Medicine. In this study, we demonstrated that *Morinda officinalis* oligosaccharides (MOO) effectively alleviated LPS-induced uterine injury in mice by reducing inflammation, oxidative stress, and apoptosis. Transcriptomic sequencing suggested the involvement of calcium signaling in MOO-mediated regulation, and in vitro experiments confirmed that MOO modulates LPS-induced inflammatory and oxidative responses in BENDs through calcium signaling.

Recent studies have highlighted MOO’s therapeutic effects on various conditions. Zhang et al. revealed that MOO alleviates depression by modulating the gut microbiota-derived serotonin metabolic pathway [14]. Additionally, MOO has been shown to activate PI3K/Akt/mTOR-mediated mitophagy to reduce neuroinflammation and mitochondrial damage during hypertension-induced depression [17]. Similarly, Qing et al. demonstrated that MOO suppresses pro-inflammatory cytokine release in an LPS-induced acute lung injury model [20]. Consistent with these prior studies, our results confirmed that MOO significantly alleviates LPS-induced endometritis in mice, a widely used model for investigating therapeutic strategies for this condition. Morphological and molecular evidence showed that MOO effectively modulates Toll-like receptor 4 (TLR4) and nuclear factor κB (RELA) signaling pathways. TLR4, a key receptor in innate immunity, is critical for recognizing pathogenic microorganisms and mediating immune responses [30]. Its role in LPS-induced mastitis and endometritis has been extensively studied, making it a focal point in related research [31,32]. TLR4 activation by LPS triggers NF-κB-mediated transcription of inflammatory cytokines. Both in vivo and in vitro, we found that MOO inhibited TLR4/RELA-mediated inflammatory cytokine release, consistent with its known anti-inflammatory effects despite differences in experimental models.

In addition, we demonstrated that MOO reduces oxidative stress, as evidenced by increased expression of antioxidative molecules such as HMOX1 and NQO1 and reduced ROS accumulation and mitochondrial dysfunction (detected by JC-1). This aligns with findings by Liu et al., who reported that MOO alleviates cyclophosphamide-induced oxidative damage in mouse testes through modulation of antioxidant pathways [33]. Although the specific markers and models differ, the protective antioxidative effects of MOO are evident across studies. Furthermore, since apoptosis of uterine epithelial cells is a critical feature of endometritis, we included apoptosis-related markers in our analysis. As anticipated, MOO exhibited significant anti-apoptotic effects, supported by molecular evidence. These findings align with prior studies demonstrating MOO’s ability to reduce neuronal apoptosis, although the earlier research primarily relied on histological assessments rather than molecular markers [34].

Calcium signaling plays a critical role in regulating inflammatory responses, with intracellular Ca^2^⁺ levels acting as a unique second messenger to modulate diverse epithelial cell functions through downstream signaling cascades [10]. Studies have demonstrated that the generation of inflammatory mediators can be triggered in various cells by Ca^2^⁺-dependent activation of transcription factors, such as NF-κB, via calcium influx [35]. For example, LPS-induced inflammation in mouse hippocampal models has been shown to require calcium signaling mediation [36]. Consistent with our findings, LPS treatment significantly enhanced calcium influx in BENDs, as evidenced by Fluo-4 fluorescence staining. Similarly, Jhamat et al. reported that LPS activates calcium signaling in fifth-generation BENDs, although their study employed higher LPS concentrations than ours [11].

Our transcriptomic and in vitro analyses demonstrate that MOO attenuates LPS-induced calcium overload in BENDs, thereby alleviating inflammation, oxidative stress, and apoptosis. To our knowledge, this is the first study to report MOO’s anti-inflammatory effects via calcium signaling modulation. Intriguingly, contrasting roles of *Morinda officinalis* components in calcium signaling have been observed: *Morinda officinalis* saponins (MOS) enhance calcium flux in human mesenchymal stem cells to promote osteogenesis [37], and *Morinda officinalis* polysaccharides elevate calcium levels in bone marrow stem cells at medium-to-high doses [38]. These divergent outcomes likely stem from differences in active components, cellular contexts, and disease-specific pathways. Specifically, MOO (oligosaccharides) differs structurally and functionally from MOS/polysaccharides, while pro-inflammatory (endometrial cells) versus pro-regenerative (stem cells) microenvironments engage distinct calcium signaling cascades. Moreover, calcium overload exacerbates endometritis but facilitates bone repair. Collectively, these findings highlight the complexity of calcium signaling pathways and their context-specific roles, underscoring the need for precise therapeutic targeting based on component chemistry, cell type, and disease pathophysiology.

Despite these promising findings, this study has limitations. First, the in vivo experiments were conducted in mice rather than dairy cows, limiting the translational relevance to bovine reproductive health. Second, while we identified that MOO suppresses calcium influx as a key mechanism for its anti-inflammatory effects, the precise molecular interactions between MOO and calcium channels or signaling regulators remain unclear. Future studies should focus on elucidating these mechanisms and validating the efficacy of MOO in animal trials using dairy cows.

## 5. Conclusions

**Figure 10:**
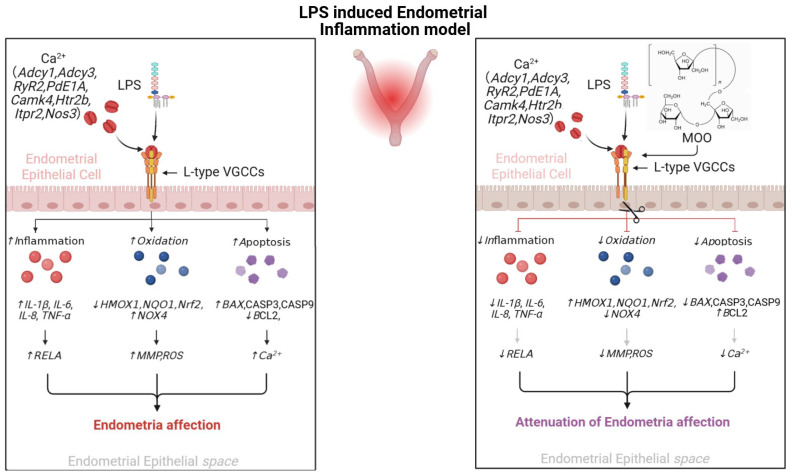
Schematic diagram of MOO’s protective effects against LPS-induced uterine epithelial cell damage. Schematic illustrating the mechanisms by which MOO alleviates inflammation, oxidative stress, and apoptosis in uterine epithelial cells through the modulation of calcium signaling.

This study highlights the anti-inflammatory and cytoprotective effects of MOO in both in vitro and in vivo models. In vivo, MOO administration in mice effectively mitigated LPS-induced uterine inflammation, as confirmed by histological improvements, downregulation of pro-inflammatory cytokines, and reduced oxidative stress markers in uterine tissues. Transcriptomic analysis further revealed that MOO treatment modulated pathways related to the calcium signaling pathway, immune responses, and cytokine–cytokine receptor interaction, supporting its broad regulatory role in maintaining endometrial homeostasis. In BENDs, MOO was shown to alleviate LPS-induced inflammation, oxidative stress, and apoptosis by inhibiting calcium influx, as evidenced by reduced intracellular calcium levels and the attenuation of inflammatory markers (Figure 10). This research will pave the way for MOO’s potential application in preventing or managing inflammation-associated reproductive disorders in livestock.

## Data Availability

The data that support the findings of this study are available from the corresponding author upon reasonable request.

## Supplementary Materials

The following supporting information can be downloaded at: https://www.mdpi.com/article/10.3390/ani15091286/s1, Table S1: Primer pairs used for Real time quantitative PCR. Figure S1. Phase Contrast Characterization of BEND Cell Morphology Under LPS-treated and MOO Intervention (200× Magnification).

## Abbreviations

The following abbreviations are used in this manuscript:

MOO	*Morinda officinalis* oligosaccharides
LPS	lipopolysaccharide
BENDs	Bovine endometrial epithelial cells
MO	*Morinda officinalis*
SPF	Specific-pathogen-free
FBS	Fetal bovine serum
LSD	Least significant difference
PCA	Principal component analysis
DEGs	Differentially expressed genes
KEGG	Kyoto Encyclopedia of Genes and Genomes
GO	Gene Ontology
MMP	Mitochondrial membrane potential
