# Supplementary material for: Morinda officinalis Oligosaccharides Protect Against LPS-Induced Uterine Damage and Endometrial Inflammation in Mice and Bovine Endometrial Epithelial Cells
Source: Animals (Basel). 2025 Apr 30;15(9):1286. doi: 10.3390/ani15091286 (PMC12071045; doi:10.3390/ani15091286)

Table 1. Primer pairs used for Real time quantitative PCR

| Gene | Forward Sequences(5´→3´) | Reverse Sequences(5´→3´) |
|---|---|---|
| *B-Nrf2* | CCCAGTCTTCACTGCTCCTC | TCAGCCAGCTTGTCATTTTG |
| *B-HMOX1* | CCTCCAAAAGCCTTGGGTCT | CAAACCACCCCTACCCTGTT |
| *B-NQO1* | CTGATTAGGCTCAAAAGGGCT | GGAGGTGGATGCTCTCTAGC |
| *B-NOX4* | ACTCTGCTGGATGACTGGAAACCA | AGAGTAAGTCTGCAAACCAGCGGA |
| *B-IL-8* | CCTCGGTTCCATGGGAGATG | AGGCACTGTTCCTCAGCTTC |
| *B-IL-6* | GCTGAATCTTCCAAAAATGGAGG | GCTTCAGGATCTGGATCAGTG |
| *B-TNF-α* | TCCAGAAGTTGCTTGTGCCT | CAGAGGGCTGTTGATGGAGG |
| *B-IL-1β* | TCCACCTCCTCTCACAGGAAA | TACCCAAGGCCACAGGAA |
| *B-BAX* | CACCAAGAAGCTGAGCGAGTGT | TCGGAAAAAGACCTCTCGGGGA |
| *B-BCL2* | TGGATGACCGAGTACCTGAA | CAGCCAGGAGAAATCAAACA |
| *B-CASP9* | AGCAAATGGTCCAGGCTTTG | ATTCTCTCGACGGACACAGG |
| *B-CASP3* | CAGCGTCGTAGCTGAACGTAA | ATCGACAGGCCATGCCAGTAT |
| *B-GAPDH* | GAAGGTCGGAGTGAACGGATTC | AAGGGGTCATTGATGGCGAC |
| *M-Camk4* | GAGAACCTCGTCCCGGATTAC | ACACAATGGATGTAGCACCCC |
| *M-Nos3* | AAGCCCGGGACTTCATCAAT | CCAAACACCAGCTCGCTCTC |
| *M-PdE1A* | CTAAAGATGAACTGGAGGGATCTTCGGAAC | TGGAGAAAATGGAAGCCCTAATTCAGC |
| *M-RyR2* | ACGGCGACCATCCACAAAG | AAAGTCTGTTGCCAAATCCTTCT |
| *M-Adcy3* | GACTGCCCTCAACCTGTACG | CCTGTCAGTGCCATTGAGCC |
| *M-Adcy1* | GTTCACAGCAGACACGATG | GGCACTGGTTGACTATGTA |
| *M-Htr2b* | ATTGCCCTCTTGACAATCATGT | GGAATAACCAGGCAGGACAC |
| *M-Itpr2* | CCTCGCCTACCACATCACC | TCACCACTCTCACTATGTCGT |
| *M-GAPDH* | TTGATGGCAACAATCTCCAC | CGTCCCGTAGACAAAATGGT |

Note: B stands for Bovine and M stands for Mouse.

Figure S1. Phase Contrast Characterization of BEND Cell Morphology Under LPS-treated and MOO Intervention (200× Magnification)
(A) Control: Confluent monolayer exhibiting characteristic epithelial cobblestone morphology
(B) LPS-Treated: Notable cell rounding and monolayer disruption
(C) LPS+MOO-L: Partial cell flattening with residual detachment
(D) LPS+MOO-M: Re-established cell-cell contacts
(E) LPS+MOO-H: Near-complete morphological recovery comparable to control

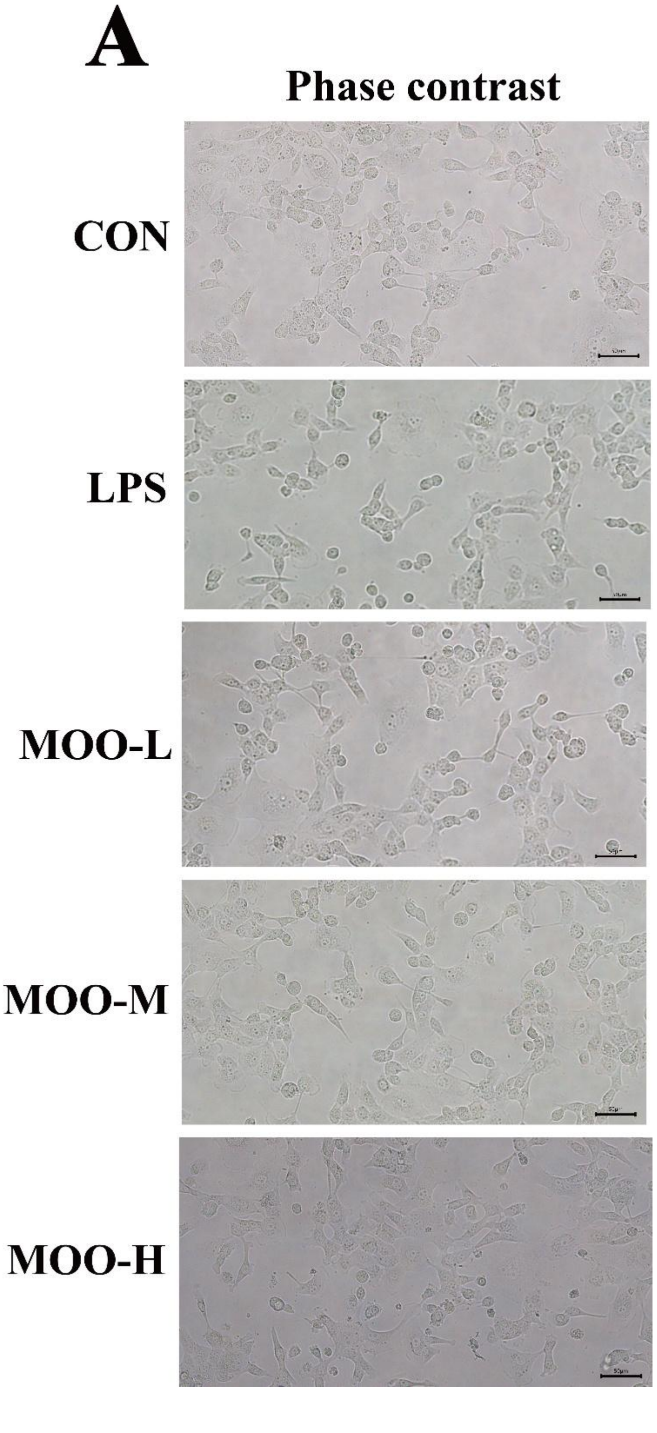

Supplement: Supplementary file 1 [file animals-15-01286-s001.zip › animals-3595264-supplementary.pdf]
